# Therapeutic limited bronchoalveolar lavage with fiberoptic bronchoscopy as a bridging procedure prior to total lung lavage in a patient with pulmonary alveolar proteinosis: a case report

**DOI:** 10.1186/s13256-015-0574-z

**Published:** 2015-04-29

**Authors:** Damith Rodrigo, Amila Rathnapala, Wijitha Senaratne

**Affiliations:** National Hospital for Respiratory Diseases, Welisara, Sri Lanka

**Keywords:** Pulmonary alveolar proteinosis, Total lung lavage, Therapeutic limited bronchoalveolar lavage

## Abstract

**Introduction:**

Therapeutic total lung lavage under general anesthesia is the current mainstay of treatment for pulmonary alveolar proteinosis, which is a rare lung disease characterized by alveolar accumulation of surfactant. Therapeutic limited bronchoalveolar lavage is considered an alternative treatment to conventional total lung lavage.

**Case presentation:**

A 61-year-old, previously healthy, Sri Lankan Moor woman presented to our facility with progressively worsening difficulty in breathing and persistent dry cough for one year. Her respiratory examination revealed bibasal fine end-inspiratory crepitations. A chest radiograph showed bilateral mid and lower zone alveolar interstitial shadows and a high-resolution computed tomography scan of her chest revealed septal thickening with ground-glass shadows more on mid and lower zones bilaterally. A diagnostic bronchoalveolar lavage fluid analysis revealed diastase-resistant protein clumps in periodic acid Schiff stain. The diagnosis was made as pulmonary alveolar proteinosis. An arterial blood gas analysis performed prior to intervention revealed a significant hypoxia (partial pressure of oxygen - 64mmHg) with alveolar-arterial gradient was 35.4mmHg. Therapeutic limited bronchoalveolar lavage was arranged and her right and her left lung were lavaged separately in two sessions done two weeks apart under local anesthesia. Our patient had significant clinical improvement and resolution of the bilateral septal thickening with minimal resolution of the ground-glass opacities in a repeat high-resolution computed tomography scan done two weeks later. Subsequently, a total lung lavage under general anesthesia was also done, which improved her dyspnea and arterial hypoxemia.

**Conclusions:**

Therapeutic limited bronchoalveolar lavage can be successfully performed as an interval bridging procedure, as a ‘prewash’, prior to conventional total lung lavage for pulmonary alveolar proteinosis.

## Introduction

Pulmonary alveolar proteinosis (PAP) is a rare lung disease characterized by alveolar accumulation of surfactant. Three main categories of PAP have been described, depending on the etiology, as autoimmune (primary or idiopathic), secondary and genetic. The disease was first described by Rosen *et al*. in 1958 [1]. Although not specific, high-resolution computed tomography (HRCT) shows a characteristic ‘crazy-paving’ pattern. Bronchoalveolar lavage (BAL) fluid analysis for periodic acid Schiff (PAS) is considered diagnostic. The current mainstay of treatment is therapeutic total lung lavage under general anesthesia in a surgical theater [2]. In case expertise with whole lung lavage is not available, such patients must be referred early to a center with facilities for total lung lavage. Another possible alternative treatment is multiple segmental or lobar lavage by fiberoptic bronchoscopy (FOB) under local anesthesia, which has been reported only in few cases in the literature [3,4]. We report our experience with limited BAL by FOB done as a bridging procedure in a patient with PAP prior to a total lung lavage.

## Case presentation

**Figure 1:**
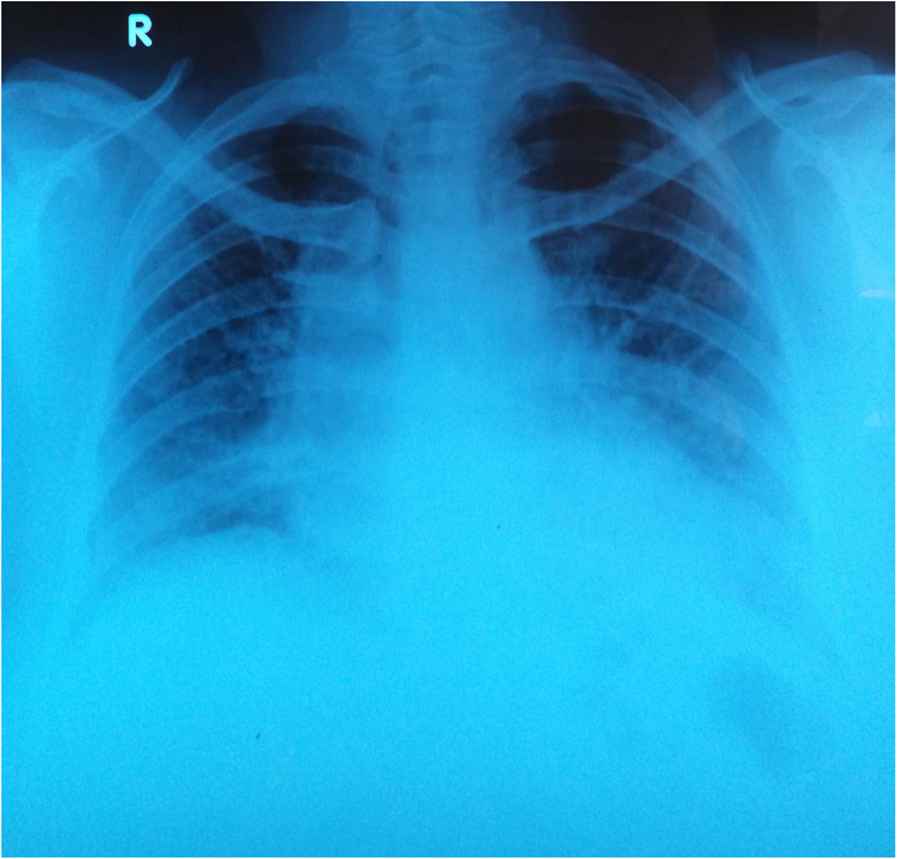
Chest radiograph on admission showing bilateral mid and lower zone alveolar interstitial shadows.

**Figure 2:**
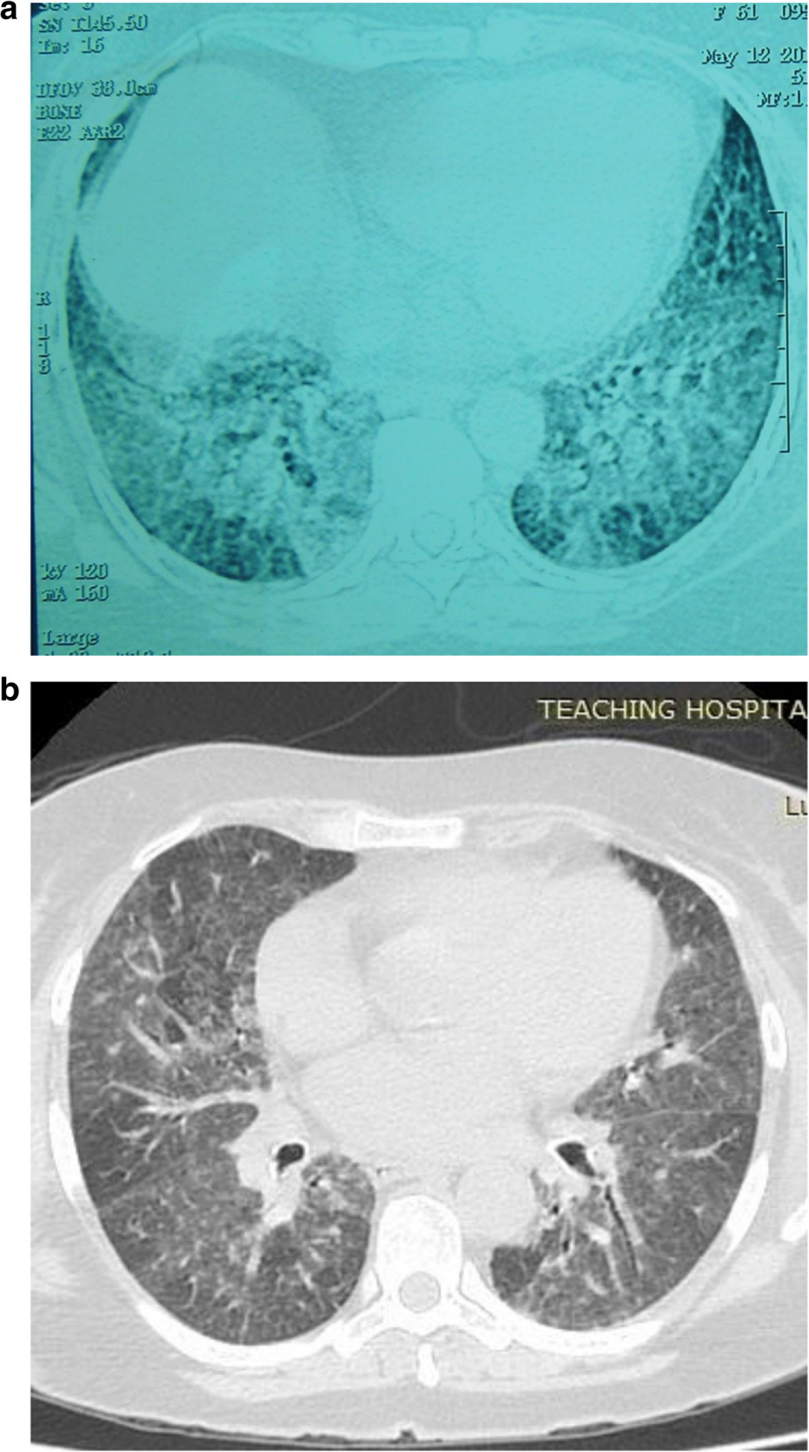
High-resolution computed tomography scans. **(a)** A high-resolution computed tomography scan of the chest (before the lavage) showing septal thickening with ground-glass shadows more on bilateral mid and lower zones. **(b)** A high-resolution computed tomography scan of the chest (after the lavage) showing significant resolution of the bilateral septal thickening with minimal resolution of ground-glass opacities.

A 61-year-old, married, previously healthy Sri Lankan Moor woman was presented to our facility with progressively worsening difficulty in breathing (modified Medical Research Council (mMRC) dyspnea scale grade 3) and persistent dry cough for one year without any history suggestive of cardiac failure. She denied chronic exposure to either organic or inorganic dust. She had no history of arthritis or connective tissues disease. Her respiratory examination revealed bilateral, bibasal fine end-inspiratory crepitations. A chest radiograph revealed bilateral mid and lower zone alveolar interstitial shadows (Figure 1). A HRCT scan of her chest showed septal thickening with ground-glass shadows more on bilateral mid and lower zones (Figure 2A). Our patient had no significant past history of cardiac illness, however, two-dimensional echocardiography was arranged and it revealed no underlying cardiac dysfunction. Her hemoglobin level was 14.5g/dL with a total leukocyte count of 9.7×10^9^/L and platelet count of 343×10^9^/L. Her renal function and liver function tests were normal. A pulmonary function test was arranged but our patient could not perform the test properly due to her symptoms. However, her six-minute walk test revealed a drop in saturation from 97% to 88% after walking 1,300 feet. A diagnostic BAL fluid analysis was carried out and it yielded a pale-yellow turbid fluid. The cytology revealed diastase-resistant protein clumps in PAS stain and was consistent with pulmonary alveolar proteinosis. The diagnosis was made as pulmonary alveolar proteinosis based on the evidence from the HRCT and positive BAL fluid cytology for PAS stain. She was investigated for an underlying cause and her serum immunoglobulins were within normal limits and retroviral screening was negative. Her rheumatoid factor and antinuclear antibodies (ANA) were normal. Her serum protein electrophoresis was also normal. An anti-granulocyte macrophage colony-stimulating factor (GM-CSF) antibody test was not performed since it was not available in the local setting.

Our patient was prepared for a therapeutic BAL and an arterial blood gas analysis was performed prior to the procedure, which revealed a significant hypoxia (partial pressure of oxygen (PaO2) - 64mmHg) while breathing room air. The alveolar-arterial gradient was 35.4mmHg. Therapeutic BAL was performed under local anesthesia without any sedation or analgesia. Local anesthesia with 2% lignocaine was given through the broncoscope as and when needed during the procedure. Our patient was connected to a pulse oxymeter and saturation was monitored throughout the procedure. One hundred percent oxygen 5L/min was used toward the latter part of the procedure when the oxygen saturation dropped below 88%. The right lung was chosen to be lavaged in the first session. A bronchoscope was passed through the right nostril and was wedged in a segmental bronchus of right upper lobe. Warm saline solution was instilled via a syringe in 50mL aliquots into each segment of the respective lobes (right upper, middle and lower) and was removed by suction into a plastic trap bottle. Small aliquots of 50mL were used for each segment and altogether 500mL warm saline was used for her entire right lung. The procedure lasted about 1hr and was stopped when the returning fluid became clear and our patient could no longer tolerate the discomfort. The chest radiograph that was performed 48hrs following the procedure revealed significant resolution of the alveolar interstitial opacities in the right lung. After two weeks, we repeated the same procedure on her left lung. Following both procedures, our patient was observed closely in an intensive care unit for few hours but in both situations the post procedure period was uneventful. A HRCT chest scan was repeated two weeks after the second lavage and was compared with the HRCT done at the beginning (Figure 2B). It showed significant resolution of the bilateral septal thickening with minimal resolution of the ground-glass opacities. An arterial blood gas analysis revealed improvement of hypoxia (PaO2 - 80mmHg). Our patient had significant clinical improvement with improvement in dyspnea (mMRC dyspnea scale grade 0 to 1) and her six-minute walk test also showed less desaturation with improvement in walk distance (1,600 feet) compared to the test done prior to the lavage. Although our patient had good clinical improvement following the procedure, the radiological improvement was minimal. Therefore, we decided to subject her to total lung lavage under general anesthesia.

## Discussion

Therapeutic total lung lavage is considered to be the gold standard treatment for PAP [2]. It should be performed under general anesthesia in a surgical theater. The endotracheal tube needs to be frequently checked and adjusted during the procedure by an experienced anesthetist. It requires at least three to four hours of theater time and a lot of manpower. Further to this, the major adverse effect of total lung lavage under general anesthesia is hypoxemia, especially toward the end of the procedure, which requires a postoperative care facility. More importantly, the patient has to be medically fit enough to undergo general anesthesia. It is often impossible to perform therapeutic total lung lavage in most patients who are newly diagnosed as PAP due to these potential complications and since the patients are usually hypoxemic and in poor clinical condition. In such cases, multiple segmental or lobar lavage by FOB has been reported as a possible alternative to whole lung lavage [3,4].

Our patient underwent limited BAL under local anesthesia in a bronchoscopy suite using an ordinary fiberoptic bronchoscope as a bridging procedure prior to conventional total lung lavage under general anesthesia. This procedure did not require general anesthesia, double-lumen endotracheal tube replacements or prolonged intensive care facility. Moreover it was safe and our patient remained hemodynamically stable throughout the procedure. This procedure showed that limited lavage can be used as a ‘prewash’ prior to conventional ‘total lung lavage’ in patients with PAP. Furthermore, this can be used a procedure for preparing the patient for conventional total lung lavage.

## Conclusions

In conclusion, limited BAL can be successfully performed as an interval bridging procedure, as a ‘prewash’, prior to conventional total lung lavage for PAP.

## Consent

Written informed consent was obtained from the patient for publication of this case report and accompanying images. A copy of the written consent is available for review by the Editor-in-Chief of this journal.

## Abbreviations

ANA: antinuclear antibodies
BAL: bronchoalveolar lavage
FOB: fiberoptic bronchoscopy
GM-CSF: granulocyte macrophage-colony stimulating factor
HRCT: high-resolution computed tomography
mMRC: modified Medical Research Council
PaO2: partial pressure of oxygen
PAP: pulmonary alveolar proteinosis
PAS: periodic acid Schiff
